# Kidney outcomes with SGLT2is for type 2 diabetes patients: does background treatment with metformin or RASis matter?

**DOI:** 10.3389/fendo.2024.1329945

**Published:** 2024-06-27

**Authors:** Kah Suan Chong, Yi-Hsin Chang, Meng-Hsuan Lin, Chien-Ning Hsu, Chi-Chuan Wang, Chih-Yuan Wang, Yun-Lin Huang, Fang-Ju Lin, Huang-Tz Ou

**Affiliations:** ^1^ Institute of Clinical Pharmacy and Pharmaceutical Sciences, College of Medicine, National Cheng Kung University, Tainan, Taiwan; ^2^ Department of Pharmacy, College of Medicine, National Cheng Kung University, Tainan, Taiwan; ^3^ School of Pharmacy, College of Medicine, National Taiwan University, Taipei, Taiwan; ^4^ Department of Pharmacy, Kaohsiung Chang Gung Memorial Hospital, Kaohsiung, Taiwan; ^5^ College of Pharmacy, Kaohsiung Medical University, Kaohsiung, Taiwan; ^6^ Graduate Institute of Clinical Pharmacy, College of Medicine, National Taiwan University, Taipei, Taiwan; ^7^ Department of Pharmacy, National Taiwan University Hospital, Taipei, Taiwan; ^8^ College of Medicine, National Taiwan University, Taipei, Taiwan; ^9^ Department of Internal Medicine, National Taiwan University Hospital, Taipei, Taiwan

**Keywords:** sodium-glucose cotransporter-2 inhibitors, metformin, renin-angiotensin system inhibitors, estimated glomerular filtration rate, kidney function

## Abstract

**Introduction:**

There is a lack of real-world evidence regarding the impact of concomitant metformin and renin-angiotensin system inhibitors (RASis) on sodium-glucose cotransporter-2 inhibitor (SGLT2i)-associated kidney outcomes. This study was aimed to investigate whether SGLT2i-associated kidney outcomes were modified by the concomitant use of metformin or RASis in patients with type 2 diabetes.

**Methods:**

SGLT2i users were identified from three electronic health record databases during May 2016 and December 2017 and categorized into those with and without concomitant use of metformin or RASis. Propensity score matching was performed to minimize baseline differences between groups. Study outcomes were mean estimated glomerular filtration rate (eGFR) change and time to 30%, 40%, and 50% eGFR reductions. A meta-analysis was performed to combine the estimates across databases.

**Results:**

After matching, there were 6,625 and 3,260 SGLT2i users with and without metformin, and 6,654 and 2,746 SGLT2i users with and without RASis, respectively. The eGFR dip was similar in SGLT2i users with and without metformin therapy, but was greater in SGLT2i users with RASis compared to those without RASis. Neither metformin nor RASi use had a significant effect on SGLT2i-associated eGFR reductions, as evidenced by the hazard ratios (95% CIs) of 30% eGFR reductions for SGLT2is with versus without metformin/RASis, namely 1.02 (0.87–1.20)/1.09 (0.92–1.31). Such findings were also observed in the outcomes of 40% and 50% eGFR reductions.

**Conclusion:**

Using metformin or RASis did not modify SGLT2i-associated kidney outcomes in type 2 diabetes.

## Introduction

Sodium-glucose cotransporter-2 inhibitors (SGLT2is) are widely used for patients with type 2 diabetes owing to their cardiovascular and nephroprotective benefits in addition to their glucose-lowering effects (1). A growing number of studies have discussed whether the kidney effects of SGLT2i therapy for type 2 diabetes patients, whose treatments commonly include multiple glucose-lowering agents (GLAs) and concomitant medications for comorbidities, could be altered by background medications (1–6). Important drugs in this regard include metformin, the first-line GLA for type 2 diabetes, and renin-angiotensin system inhibitors (RASis), the most commonly prescribed antihypertensive agents in this population (7).

Evidence regarding the impact of concomitant therapies on the kidney effects of SGLT2is remains uncertain. Some studies suggested that major kidney outcomes (e.g., reduced risk of worsening nephropathy) of SGLT2i therapy were independent of baseline metformin use (1, 2), whereas others found that such risk may be greatly reduced among SGLT2i users without metformin compared to those with metformin (3). In addition, the magnitude of the estimated glomerular filtration rate (eGFR) dip following SGLT2i initiation may be reduced in SGLT2i users taking metformin (4). It has been suggested that SGLT2i users who received concomitant RASi therapy experience an enhanced response to SGLT2i therapy (4); however, there is no evidence that the use of concomitant RASi medication can modify the composite kidney outcome of SGLT2i therapy (5, 6).

These conflicting findings regarding the effects of background treatments on SGLT2i-associated kidney outcomes may be attributed to methodology limitations and differences in study designs. Specifically, most of the studies that analyzed the effects of background treatments were *post-hoc* analyses on subsets of trial participants that were not primarily designed to investigate the impact of these treatments (2, 3, 5, 6, 8–10). Patient characteristics were often imbalanced between treatment groups and not fully adjusted in the analyses (2, 3, 5, 6, 10). Only one real-world study has examined the kidney effects of SGLT2i use under different background therapies (4); however, there is uncertainty in the between-group comparability at the patient baseline. Against this background, we sought to utilize large-scale, population-based data obtained from multiple medical institutions in Taiwan to determine whether SGLT2i-associated kidney outcomes were modified by background metformin or RASi medications, the most commonly used glucose-lowering and antihypertensive agents, respectively, among patients with type 2 diabetes in clinical practice (7, 11, 12). Considering the limited available evidence (1, 2), we hypothesized that the concomitant drugs would not substantively impact the kidney outcomes associated with SGLT2i therapy.

## Materials and methods

### Data source

The present study utilized electronic health records (EHRs) obtained from three healthcare delivery systems in Taiwan, namely National Taiwan University Hospital (NTUH), National Cheng Kung University Hospital (NCKUH), and Chang Gung Memorial Hospital (CGMH). Individual-level data of patients with type 2 diabetes derived from these databases were transformed into a common data model for analysis. Details of these study healthcare systems and the common data model transformation are described elsewhere (7, 13). This study was approved by the Research Ethics Committees of the study hospitals (NTUH: 201808029RSA, CGMH: 201900899B0C602, and NCKUH: A-ER-108–097). Since all analyses were conducted using retrospective data with de-identified patient-level records, an exemption for informed consent by individual patients was granted by the Research Ethics Committees.

### Cohort identification and follow-up

Because the first SGLT2i drug (i.e., dapagliflozin) was first reimbursed by the National Health Insurance (NHI) in Taiwan in May 2016, new users of SGLT2is were defined as patients who received their first SGLT2i prescription between May 1, 2016 and December 31, 2017. SGLT2i users were further classified into groups based their baseline concomitant use of metformin or RASis. The index date was defined as the first date of the combination of SGLT2is and metformin or RASis or the date of initiation of SGLT2is if no combination was used. Other inclusion criteria were an age of 18 years or older, at least two pre-index eGFR measurements [the latest pre-index eGFR measurement was required to be within 180 days prior to the index date with an earlier measurement at least 180 days apart (14)], and at least one eGFR measurement after the index date. Patients diagnosed with type 1 diabetes, gestational diabetes, or without medical records for at least one year before the index date were excluded. In addition, patients whose last pre-index eGFR value was less than 30 mL/min/1.73 m^2^ were excluded because the use of metformin is contraindicated in individuals with severe chronic kidney disease. The eGFR value was calculated using the Modification of Diet in Renal Disease equation: 175 × serum creatinine (SCr) (mg/dL)^–1.154^ × age (years)^–0.203^ × 0.742 (if female) (15).

Patients were observed from the index date until the study outcome occurrence, last encounter date in the EHR databases, death, or the end of the study period (i.e., December 2017 for CGMH and NTUH, and July 2018 for NCKUH), whichever came first (i.e., intention-to-treat [ITT] analytic approach).

### Study covariates and outcome measures

In addition to age and sex, a series of patient comorbidities and medications at baseline were measured and adjusted in the analysis. Specifically, patient comorbidities, including cardiovascular diseases (i.e., ischemic heart disease, heart failure, atrial fibrillation, stroke, peripheral artery disease, and transient ischemic attack), diabetes-related complications (i.e., diabetic retinopathy, neuropathy, and nephropathy), and other chronic diseases (i.e., chronic kidney disease and hypertension), were defined based on all available diagnosis records in the EHR databases before the index date. Patient hemoglobin A1c (HbA1c), eGFR level, eGFR change (measured as slope) (14), number of pre-index eGFR measurements, prior use of non-index GLAs, and other concurrent medications (i.e., hypertensive drugs, statins, aspirin, and antiplatelets) were measured in the year prior to the index date. Patient frailty status was determined based on the occurrence of a hospital stay of three or more consecutive days in the year prior to the index date. In addition, the type of SGLT2i (i.e., dapagliflozin or empagliflozin in our study period) and the quarter of the index date were measured as study covariates.

In the present study, we compared kidney outcomes between SGLT2i users with and without the background medications of interest (i.e., metformin, RASis). There were two kidney outcomes of interest, namely the mean eGFR change in the year following SGLT2i administration and the time to 30%, 40% and 50% eGFR reductions following SGLT2i initiation (7, 14). The deterioration status of kidney function (e.g., a 30% eGFR reduction) was confirmed using at least one follow-up eGFR measurement.

### Statistical analyses

**Figure 1:**
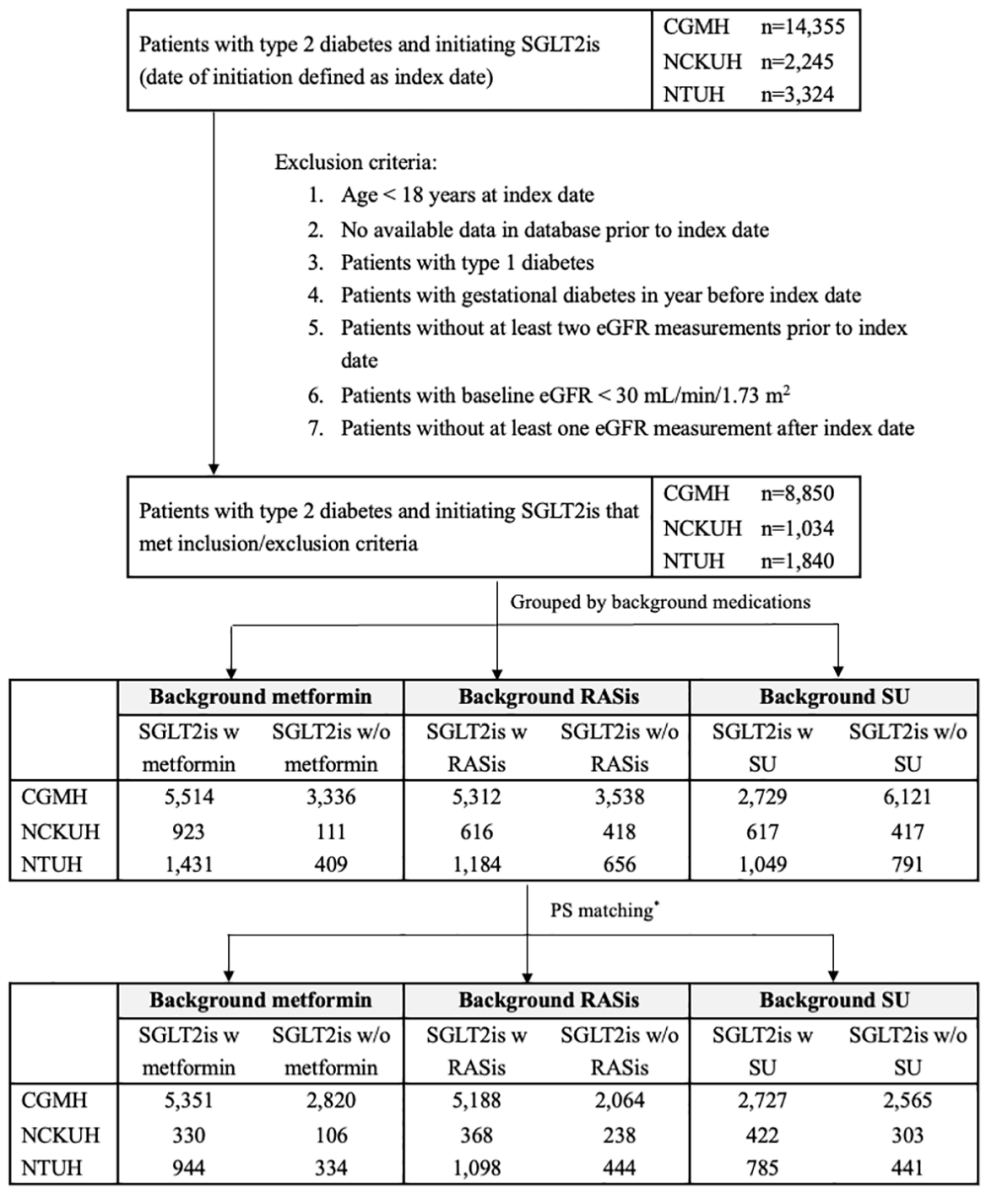
Flow chart of cohort identification in three health care delivery systems. CGMH, Chang Gung Memorial Hospital; eGFR, estimated glomerular filtration rate; NCKUH, National Cheng Kung University Hospital; NTUH, National Taiwan University Hospital; RASis, renin-angiotensin system inhibitors; SGLT2is, sodium glucose cotransporter-2 inhibitors; SU, sulfonylurea; w, with; w/o, without. ^*^ PS matching was performed at a 4:1 ratio for SGLT2is users with and without background medications in the three health care delivery systems, except for SGLT2i users with and without background RASis/SU, who were matched at a 2:1 ratio at NCKUH. Patients may have received more than one of the background therapies under investigation and thus could be represented in more than one of the comparisons.

To fairly compare SGLT2i users with and without background medications of interest, propensity score (PS) matching with the greedy nearest-neighbor method and a caliper of width equal to 0.25 of the standard deviation of the logit of the PS was performed to determine the between-group comparability at baseline (16, 17). Specifically, a logistic regression model that comprised the aforementioned covariates was conducted to estimate the PS of each patient. SGLT2i users with and without the background medication of interest were matched at a 4:1 or 2:1 ratio within each healthcare delivery system (see **Figure 1**) given no additional gain in precision when matching more than 4 controls (18). The between-group difference in baseline characteristics before and after matching was evaluated using the standardized mean difference (SMD). The average treatment effect in the treated (ATT) weights generated from the n:1 ratio matching was incorporated into the balance assessment (i.e., SMD testing) and outcome analysis (18).

We calculated the changes in eGFR values after the index date in the PS-matched cohorts. Cox proportional hazards modeling was applied to estimate the hazard ratios (HRs) with 95% CIs for 30%, 40%, and 50% eGFR reductions between SGLT2i users with and without background medications. Meta-analysis approaches were carried out to aggregate the HRs with corresponding 95% CIs of the study outcomes obtained from each healthcare delivery system. Given the possibility of heterogeneity across medical institutions, a generic inverse variance weighting with random-effects model (19) was adopted in the analysis. Forest plots are used to illustrate the results of institution-specific and overall pooled HRs (with their 95% CIs). The meta-analysis was conducted using the software RevMan 5 (Nordic Cochrane Centre, Copenhagen, Denmark).

Several sensitivity analyses were conducted to corroborate the robustness of the study findings. First, the analysis was re-run based on a strict on-treatment (sOT) analytic approach where patients were followed up until the occurrence of a study outcome, discontinuation of index medication (i.e., SGLT2is or background medications of interest), switch to or addition of other GLAs, last encounter date in the EHR database, death, or the last date of the study period, whichever came first. Second, sulfonylureas (SU), which has a neutral effect on patient kidney function (3, 4), was chosen as a negative control exposure for a background medication of interest. Third, we applied a stricter definition for eGFR reduction based on at least two consecutive eGFR measurements during the follow-up. All statistical analyses were performed using the software SAS version 9.4 (SAS Institute, Cary, NC).

## Results

There were 11,724 new users of SGLT2is in the overall study population, which comprised 8,850, 1,840, and 1,034 patients from CGMH, NTUH, and NCKUH, respectively (**Figure 1**). After PS matching, there were 6,625 and 3,260 SGLT2i users with and without metformin, 6,654 and 2,746 SGLT2i users with and without RASis, and 3,934 and 3,309 SGLT2i users with and without SU, respectively (**Table 1**).

**Table 1:** Baseline characteristics of overall study cohort after propensity score matching, grouped by use of background medications (metformin, RASis, and SU) with SGLT2is.

Characteristics	Background metformin		Background RASis		Background SU	
	SGLT2i users w metformin	SGLT2i users w/o metformin	SGLT2i users w RASis	SGLT2i users w/o RASis	SGLT2i users w SU	SGLT2i users w/o SU
	n=6,625	n=3,260	n=6,654	n=2,746	n=3,934	n=3,309
Age (years), mean ± SD	60.10 ± 11.26	60.51 ± 11.51	61.39 ± 11.01	60.96 ± 11.56	61.25 ± 11.10	60.94 ± 11.30
Male	3,891 (58.73)	1,919 (58.87)	3,926 (59.00)	1,663 (60.56)	2,307 (58.64)	1,959 (59.20)
Baseline HbA1c (%), mean ± SD	8.58 ± 1.44	8.77 ± 1.46	8.60 ± 1.47	8.70 ± 1.47	8.80 ± 1.33	8.77 ± 1.60
Baseline HbA1c (mmol/mol), mean	70	72	70	72	73	72
Baseline eGFR (ml/min/1.73 m^2^), mean ± SD	86.21 ± 25.68	84.57 ± 27.63	82.36 ± 24.99	86.52 ± 26.75	84.47 ± 27.25	84.93 ± 26.70
eGFR>90, n (%)	2,683 (40.50)	1,257 (38.56)	2,303 (34.61)	1,138 (41.44)	1,492 (37.93)	1,270 (38.38)
60<eGFR ≤ 90, n (%)	2,923 (44.12)	1,381 (42.36)	3,071 (46.15)	1,156 (42.10)	1,696 (43.11)	1,428 (43.16)
45<eGFR ≤ 60, n (%)	809 (12.21)	451 (13.83)	984 (14.79)	333 (12.13)	539 (13.70)	454 (13.72)
eGFR ≤ 45, n (%)	210 (3.17)	171 (5.25)	296 (4.45)	119 (4.33)	207 (5.26)	157 (4.74)
eGFR change in year before index date, mean ± SD (ml/min/1.73 m^2^)	-1.58 ± 13.20	-1.45 ± 13.21	-1.65 ± 12.62	-1.31 ± 13.64	-1.41 ± 12.86	-1.44 ± 13.08
Number of pre-index-date eGFR measurements, mean ± SD	6.42 ± 4.23	6.80 ± 4.45	6.48 ± 4.14	6.87 ± 4.64	6.35 ± 3.90	6.35 ± 3.83
History of microvascular disease, n (%)	3,155 (47.62)	1,670 (51.23)	3,226 (48.48)	1,348 (49.09)	1,899 (48.27)	1,591 (48.08)
History of cardiovascular disease, n (%)						
Ischemic heart disease	2,105 (31.77)	1,045 (32.06)	2,343 (35.21)	959 (34.92)	1,197 (30.43)	1,048 (31.67)
Heart failure	622 (9.39)	355 (10.89)	755 (11.35)	305 (11.11)	371 (9.43)	310 (9.37)
Atrial fibrillation	254 (3.83)	145 (4.45)	310 (4.66)	142 (5.17)	165 (4.19)	134 (4.05)
Stroke	883 (13.33)	478 (14.66)	1,028 (15.45)	397 (14.46)	566 (14.39)	479 (14.48)
Peripheral artery disease	297 (4.48)	193 (5.92)	370 (5.56)	159 (5.79)	183 (4.65)	160 (4.84)
Transient ischemic attack	202 (3.05)	109 (3.34)	230 (3.46)	94 (3.42)	122 (3.10)	105 (3.17)
Chronic kidney disease, n (%)	575 (8.68)	334 (10.25)	719 (10.81)	313 (11.40)	486 (12.35)	380 (11.48)
Hypertension, n (%)	4,945 (74.64)	2,454 (75.28)	6,028 (90.59)	2,180 (79.39)	2,902 (73.77)	2,471 (74.68)
Baseline status of frailty, n (%)	829 (12.51)	443 (13.59)	890 (13.38)	395 (14.38)	449 (11.41)	415 (12.54)
Medication history of glucose-lowering agent in year prior to index date, n (%)						
Metformin	NA^*^	NA^*^	5,284 (79.41)	2,150 (78.30)	3,509 (89.20)	2,929 (88.52)
SU	3,358 (50.69)	1,144 (35.09)	3,142 (47.22)	1,333 (48.54)	NA^*^	NA^*^
Meglitinide	182 (2.75)	77 (2.36)	190 (2.86)	80 (2.91)	54 (1.37)	56 (1.69)
DPP-4 inhibitor	4,980 (75.17)	2,380 (73.01)	4,942 (74.27)	1,983 (72.21)	3,079 (78.27)	2,592 (78.33)
Thiazolidinedione	1,853 (27.97)	715 (21.93)	1,689 (25.38)	714 (26.00)	1,228 (31.22)	941 (28.44)
GLP-1 receptor agonist	181 (2.73)	78 (2.39)	185 (2.78)	69 (2.51)	104 (2.64)	85 (2.57)
Acarbose	1,382 (20.86)	746 (22.88)	1,524 (22.90)	615 (22.40)	974 (24.76)	793 (23.96)
Insulin	1,452 (21.92)	831 (25.49)	1,472 (22.12)	660 (24.03)	734 (18.66)	673 (20.34)
Other medications, n (%)						
Loop diuretic	460 (6.94)	292 (8.96)	568 (8.54)	235 (8.56)	315 (8.01)	258 (7.80)
Thiazide diuretic	216 (3.26)	102 (3.13)	242 (3.64)	66 (2.40)	148 (3.76)	109 (3.29)
RASis	4,330 (65.36)	2,094 (64.23)	NA^*^	NA^*^	2,571 (65.35)	2,163 (65.37)
CCB	1,576 (23.79)	780 (23.93)	1,969 (29.59)	679 (24.73)	1,002 (25.47)	773 (23.36)
β-blocker	2,434 (36.74)	1,212 (37.18)	2,896 (43.52)	1,001 (36.45)	1,407 (35.77)	1,211 (36.60)
Aldosterone antagonists	273 (4.12)	150 (4.60)	292 (4.39)	141 (5.13)	169 (4.30)	129 (3.90)
Statins	3,832 (57.84)	1,840 (56.44)	3,851 (57.87)	1,588 (57.83)	2,246 (57.09)	1,905 (57.57)
Aspirin	2,148 (32.42)	1,035 (31.75)	2,497 (37.53)	889 (32.37)	1,265 (32.16)	1,095 (33.09)
Antiplatelet agent	579 (8.74)	321 (9.85)	681 (10.23)	259 (9.43)	328 (8.34)	304 (9.19)
Anticoagulant	189 (2.85)	104 (3.19)	193 (2.90)	98 (3.57)	111 (2.82)	98 (2.96)
SGLT2i category, n (%)						
Dapagliflozin	2,964 (44.74)	1,497 (45.92)	2,977 (44.74)	1,279 (46.58)	1,916 (48.70)	1,542 (46.60)
Empagliflozin	3,661 (55.26)	1,763 (54.08)	3,677 (55.26)	1,467 (53.42)	2,018 (51.30)	1,767 (53.40)
Quarter of SGLT2i initiation, n (%)						
2^nd^ quarter, 2016	600 (9.06)	335 (10.28)	612 (9.20)	248 (9.03)	373 (9.48)	327 (9.88)
3^rd^ quarter, 2016	1,690 (25.51)	838 (25.71)	1,627 (24.45)	644 (23.45)	994 (25.27)	863 (26.08)
4^th^ quarter, 2016	1,338 (20.20)	614 (18.83)	1,358 (20.41)	520 (18.94)	795 (20.21)	652 (19.70)
1^st^ quarter, 2017	1,075 (16.23)	527 (16.17)	1,087 (16.34)	474 (17.26)	638 (16.22)	533 (16.11)
2^rd^ quarter, 2017	891 (13.45)	463 (14.20)	921 (13.84)	385 (14.02)	529 (13.45)	438 (13.24)
3^rd^ quarter, 2017	728 (10.99)	329 (10.09)	730 (10.97)	309 (11.25)	387 (9.84)	323 (9.76)
4^th^ quarter, 2017	303 (4.57)	154 (4.72)	319 (4.79)	166 (6.05)	218 (5.54)	173 (5.23)

CCB, calcium channel blocker; DPP-4 inhibitor, dipeptidyl peptidase 4 inhibitor; eGFR, estimated glomerular filtration rate; GLP-1 receptor agonist, glucagon-like peptide 1 receptor agonist; HbA1c, hemoglobin A1c; NA, not applicable; RASis, renin-angiotensin system inhibitors; SD, standard deviation; SGLT2is, sodium glucose cotransporter-2 inhibitors; SU, sulfonylurea; w, with; w/o, without.

^*^This variable was not measured in the cohort and was not included in the estimation of propensity score and matching.

Patients may have received more than one of the background therapies under investigation and thus could be represented in more than one of the comparisons.

### Effect of metformin on SGLT2i-associated eGFR responses

After matching, the baseline patient characteristics were comparable between the 6,625 SGLT2i users with metformin (e.g., mean age 60.10 years; mean baseline HbA1c 8.58% [70 mmol/mol]; mean baseline eGFR 86.21 ml/min/1.73 m^2^; 74.64% hypertension) and the 3,260 users without metformin (e.g., 60.51 years; 8.77% [72 mmol/mol]; 84.57 ml/min/1.73 m^2^; 75.28%), as supported by all SMD less than 0.1 (**Table 1**; **Supplementary Table S1**). Statins and RASis were the most frequently prescribed medications in addition to GLAs in the year before or at the index date. Empagliflozin was the most frequently prescribed SGLT-2i during the study period (accounting for 54.1–55.3% of all SGLT-2i users).

**Figure 2:**
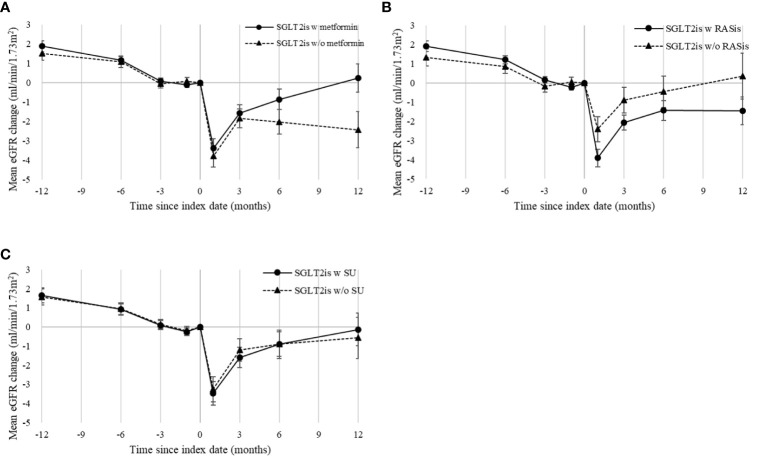
Mean eGFR changes following SGLT2i initiation in overall propensity-score-matched cohort, grouped by use of background medications: **(A)** metformin, **(B)** RASis, and **(C)** SU. eGFR, estimated glomerular filtration rate; RASis, renin-angiotensin system inhibitors; SGLT2is, sodium glucose cotransporter-2 inhibitors; SU, sulfonylurea; w, with; w/o, without. Index date refers to the date of SGLT2i therapy initiation or the first date of combination of SGLT2is and background medication of interest. Mean changes in eGFR are plotted with standard error bars.

**Figure 3:**
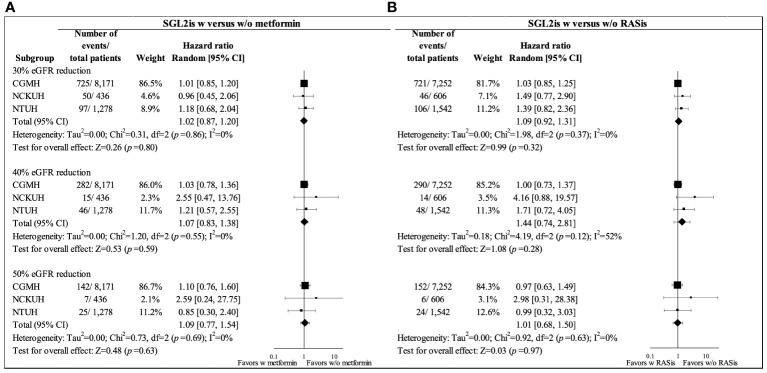
Forest plots for 30%, 40%, and 50% eGFR reductions in propensity-score-matched SGLT2i users with and without background medications in overall study cohort from three health care delivery systems (CGMH, NCKUH, and NTUH) (intention-to-treat analysis): **(A)** metformin and **(B)** RASis. CGMH, Chang Gung Memorial Hospital; eGFR, estimated glomerular filtration rate; NCKUH, National Cheng Kung University Hospital; NTUH, National Taiwan University Hospital; RASis, renin-angiotensin system inhibitors; SGLT2is, sodium glucose cotransporter-2 inhibitors; w, with; w/o, without.

In the primary analysis using the ITT analytic approach, the mean follow-up for eGFR reduction among the users with and without metformin background medication was 365.19 and 365.05 days, respectively. **Figure 2A** shows similar trends of the eGFR changes for the two treatment groups in the overall study population. An initial eGFR dip was observed in the first month after SGLT2i initiation in both patients with and without metformin (with mean eGFR changes of -3.4 and -3.8 ml/min/1.73 m^2^, respectively); eGFR then recovered at 3 months. Cox regression analysis using the ITT analytic approach showed no significant difference in eGFR reductions between metformin users and non-users after SGLT2is initiation, with HRs (95% CIs) of 1.02 (0.87–1.20), 1.07 (0.83–1.38), and 1.09 (0.77–1.54) for 30%, 40%, and 50% eGFR reductions, respectively (**Figure 3**).

### Effect of RASis on SGLT2i-associated eGFR responses

The baseline characteristics were generally similar between SGLT2i users with and without RASis after PS matching (e.g., mean age 61.39 versus 60.96 years; mean baseline HbA1c 8.60% [70 mmol/mol] versus 8.70% [72 mmol/mol]; mean baseline eGFR 82.36 versus 86.52 ml/min/1.73 m^2^), except for a higher prevalence of hypertension and more use of β-blockers among the RASi users compared to non-RASi users (i.e., 90.59% versus 79.39% with hypertension, 43.52% versus 36.45% using β-blockers), as supported by all SMD less than 0.1 (**Table 1**; **Supplementary Table S2**). All characteristics were balanced after incorporating the ATT matching weights into the SMD analysis.

The mean follow-up for eGFR reduction among the users with and without RASi background medication was 365.34 and 361.46 days, respectively, under the ITT analytic approach. **Figure 2B** shows a greater eGFR dip at one month following SGT2i therapy among patients with RASis compared to those without RASis (-3.92 versus -2.41 ml/min/1.73 m^2^) among the overall study population. However, no significant difference in eGFR reduction between RASis users and non-users after SGLT-2is initiation was found, with HRs (95% CIs) of 1.09 (0.92–1.31), 1.44 (0.74–2.81), and 1.01 (0.68–1.50) for 30%, 40%, and 50% eGFR reductions, respectively (**Figure 3**).

### Sensitivity analyses

The sOT analysis results (**Supplementary Figure S2**) are consistent with the primary analysis findings based on the ITT analytic approach. In addition, the analysis using SU as a background medication indicated no significant effect of SU on SGLT2i-associated eGFR responses, including the mean changes (**Figure 2C**) and the risk of eGFR reduction (**Supplementary Figure S1** for ITT analytic approach; **Supplementary Figures S2–S3** for sOT analytic approach). Moreover, the analysis of the eGFR reduction that was re-run using the stricter definition (i.e., at least two follow-up eGFR reductions) showed consistent results (**Supplementary Figures S3** and **S4**) with the main analysis findings.

## Discussion

To our best knowledge, this was the largest multi-institutional study (with almost 12,000 patients) to assess the possible effects of background medication on SGLT2i-associated eGFR responses in the real world, including mean eGFR changes and the risks of 30%, 40%, and 50% eGFR reductions. With careful adjustment of all potential confounders through PS matching, we found no significant effect of metformin or RASis on the eGFR responses to SGLT2i therapy. The eGFR dip following SGLT2i therapy was generally comparable between patients with and without metformin; the change was slightly greater in patients with RASis compared to those without RASis. The SGLT2i-associated effect on the risk of eGFR reduction was independent of metformin or RASi use. These results are substantiated by a series of sensitivity analyses (i.e., sOT analytic approach using SU as negative control exposure and with a stricter definition of eGFR reduction). Hence, our results indicate that combining SGLT2i therapy with metformin or RASis does not compromise the kidney outcomes of SGLT2i therapy (e.g., reducing the risk of eGFR reduction). In addition, we did not find any evidence to suggest that these combinations lead to an increased incidence of adverse kidney events. Therefore, using multiple therapies (i.e., SGLT2i therapy with metformin or RASis) should still be considered and is recommended for maximizing the benefits of glycemic control and kidney protection in individuals with type 2 diabetes.

Previous research on the effect of metformin on the renal hemodynamic effects of SGLT2is has yielded conflicting results. The impact of metformin treatment itself on renal function in individuals with type 2 diabetes has been inconsistent; some studies showed kidney benefits (20, 21) while others indicated the worsening of renal function (22, 23). Moreover, several studies suggested that metformin may facilitate nitric oxide production by stimulating of AMP-activated protein kinase, thereby attenuating the tubuloglomerular feedback action induced by SGLT2is and reducing eGFR responses to SGLT2i therapy (24). While a recent observational study found a smaller reduction in eGFR initial responses to SGLT2i therapy among metformin users compared to non-users (4), a *post-hoc* analysis of the EMPA-REG OUTCOME trial revealed a smaller empagliflozin-induced reduction in the risk of nephropathy among patients with metformin than those without metformin (3). These findings conflict with the results of the present study and a meta-analysis of SGLT2i trials, which showed that SGLT2i therapy provided consistent kidney benefits regardless of concurrent metformin use (1). Differences in study designs, patient characteristics (e.g., different cardiovascular risks), kidney outcome measures (e.g., eGFR responses versus hard kidney events), and analytic methods used to ensure study validity (e.g., adjustment for between-group imbalances) may explain the inconclusive evidence across studies, and thus caution should be made while directly comparing study findings. The combination of SGLT2i and RASi therapies may lead to a synergistic effect of vasoconstriction of the afferent arteriole and vasodilation of the efferent arteriole, which reduces the intraglomerular pressure in patients with diabetic kidney diseases (25, 26). Therefore, the combination of these two drugs is commonly recommended for these patients (25). Since background RASi therapy may augment the renal hemodynamic effect of SGLT2is, patients taking RASis are expected to experience a greater initial eGFR reduction (i.e., dip) following SGLT2i therapy than those without RASis (4, 6, 27). The analysis of the EMPA-REG OUTCOME trial showed that the background use of RASis significantly modified the SGLT-2i-induced early eGFR changes (i.e., amplifying the eGFR dip following SGLT-2i therapy) over 4 weeks of follow-up since treatment initiation (*P*_interaction_ = 0.0003) (6). Our study showed a similar enhanced renal hemodynamic effect attributable to the combination with RASi therapy (i.e., -3.92 mL/min/1.73 m^2^ of initial eGFR change during the first month of SGLT2i therapy in patients with RASis versus -2.41 mL/min/1.73 m^2^ in those without RASis), as did a previous study of real-world type 2 diabetes patients [i.e., -4.5 mL/min/1.73 m^2^ of initial eGFR change in the RASi users versus -2.8 mL/min/1.73 m^2^ of change in non-users in the first three months (4)]. Although RASi use may result in a greater initial eGFR change, the *post-hoc* analysis of the DECLARE-TIMI-58 trial demonstrated that baseline RASi use did not modify the kidney benefits associated with SGLT2i therapy (*P*_interaction_ = 0.16) (5). Similarly, the *post-hoc* analysis of the EMPA-REG OUTCOME trial revealed no significant modification effect of background RASi use on incident or worsening nephropathy (*P*_interaction_ = 0.0578), doubling of serum creatinine, initiation of renal replacement therapy, and death from renal disease (*P*_interaction_ = 0.6118) associated with SGLT2i therapy (6). Consistent with these findings, the present real-world study found that the renal benefits of SGLT2i therapy, such as lowering the risk of eGFR reduction by 30% or more, were not influenced by baseline RASi use. However, the long-term impact of RASi use on SGLT2i-associated kidney outcomes requires further investigation.

Our study has several inherent limitations that need to be acknowledged. First, similar to other observational studies, the possibility of residual or unmeasured confounding in our analyses could not be entirely ruled out. However, we attempted to minimize this concern by controlling for proxies. Specifically, we adjusted the use of GLAs and the history of diabetes-related complications to account for diabetes severity or glycemic control. We further performed PS estimation and matching within each health care delivery system to account for potential variations in clinical practice. The analysis using SU as a negative control exposure supports the validity of our study procedures. Second, our study mainly focused on metformin and RASis due to their clinical importance and frequent use in treating patients with type 2 diabetes. Future research should explore other GLAs and concomitant drugs. Third, our analyses were limited to short-term eGFR responses. Additional studies are needed to investigate the long-term impact of these drugs on hard kidney outcomes. However, since an eGFR reduction of 30% or more has been associated with adverse kidney events such as the doubling of serum creatinine levels and progression to end-stage kidney disease (11), our findings may still provide insight into the forecasting of severe kidney outcomes. Fourth, due to the stringent data use policies and the imperative need to uphold individual record privacy within each institution, the integration of individual-level data across different institutions was not feasible. Fifth, due to the data availability of each institution, the present study involving multi-centers was limited to utilize the data mainly from 2016 to 2017. Hence, future studies with the latest data are warranted to confirm our findings. Sixth, it is important to consider the potential influence of competing risk of death on the study results. Nonetheless, the concern is anticipated to be mitigated by the composition of our study cohort, which mainly consists of less severe/frail patient populations and by the relatively short follow-up of less than one year. Future research applying statistical analyses with adjusting competing risk of death remains warranted to corroborate our findings obtained using traditional survival analyses. Seventh, the possibility of heterogeneity in individual patient-level data across multiple institutions could not be eliminated. Nonetheless, a generic inverse variance method with random effects in the meta-analysis was employed to minimize this concern and ensure the validity of our results. Lastly, the present study was aimed to analyze the kidney outcomes of SGLT2i therapies and stratified by the presence of background medication of interest (e.g., metformin). So, we did not further investigate the adverse events following these therapies (e.g., metformin-associated acidosis) (28), which however might be of clinical interest for the use of these medications.

In conclusion, our study found that the kidney outcomes of SGLT2i therapy were not affected by the concurrent use of metformin or RASis, which are frequently prescribed medications in patients with type 2 diabetes. Therefore, the combination of SGLT2is with metformin or RASis remain important for improving the glycemic control and kidney outcomes for type 2 diabetes patients who require multiple GLAs and are at risk of progressive kidney diseases, respectively. Further research is needed to verify the long-term kidney outcomes for these combinations.

## Author's note

Parts of this study were presented in abstract form at ISPE’s 14th Asian Conference on Pharmacoepidemiology (ACPE), October 21–23, 2022.

## Data availability statement

The datasets presented in this article are not readily available because the individual-level data were not available due to the data privacy of each institution. Requests to access the datasets should be directed to huangtz@mail.ncku.edu.tw.

## Ethics statement

The studies involving humans were approved by Research Ethics Committees of National Taiwan University Hospital, Research Ethics Committees of National Cheng Kung University Hospital, Research Ethics Committees of Chang Gung Memorial Hospital. The studies were conducted in accordance with the local legislation and institutional requirements. The participants provided their written informed consent to participate in this study.

## Author contributions

KC: Writing – original draft. Y-HC: Writing – original draft. M-HL: Writing – review & editing. C-NH: Writing – review & editing. C-CW: Writing – review & editing. C-YW: Writing – review & editing. Y-LH: Writing – review & editing. F-JL: Writing – review & editing. H-TO: Writing – review & editing.
