# Supplementary material for: Kidney outcomes with SGLT2is for type 2 diabetes patients: does background treatment with metformin or RASis matter?
Source: Front Endocrinol (Lausanne). 2024 Jun 27;15:1329945. doi: 10.3389/fendo.2024.1329945 (PMC11236716; doi:10.3389/fendo.2024.1329945)
Supplement: Supplementary file 1 [file DataSheet_1.docx]

**SUPPLEMENTARY APPENDIX**

**List of all centers and participating investigators contributing to this analysis**

**National Cheng Kung University Hospital (NCKUH)**

Huang-Tz Ou (principal investigator, PI), Kah-Suan Chong, Yi-Hsin Chang

**National Taiwan University Hospital (NTUH)**

Fang-Ju Lin (PI), Meng-Hsuan Lin, Chi-Chuan Wang, Chih-Yuan Wang, Yun-Lin Huang

**Chang Gung Memorial Hospital (CGMH)**

Chien-Ning Hsu (PI), Hsiao-Ching Kuo

**Supplementary Table 1.** Baseline characteristics of study cohort after propensity score matching in each institution, grouped by use of metformin with SGLT2is

| Characteristics | | CGMH | |  | NTUH | |  | NCKUH | |
| --- | --- | --- | --- | --- | --- | --- | --- | --- | --- |
|  |  | SGLT2i users w metformin | SGLT2i users  w/o metformin |  | SGLT2i users w metformin | SGLT2i users  w/o metformin |  | SGLT2i users w metformin | SGLT2i users  w/o metformin |
|  |  | n=5,351 | n=2,820 |  | n=944 | n=334 |  | n=330 | n=106 |
| Age (years), mean ± SD | | 59.41 ± 11.29 | 59.97 ± 11.58 |  | 63.89 ± 11.18 | 64.93 ± 10.75 |  | 60.47 ± 11.04 | 61.11 ± 12.11 |
| Male | | 2,293 (42.85) | 1,659 (58.83) |  | 565 (59.85) | 199 (59.58) |  | 186 (56.36) | 61 (57.55) |
| Baseline HbA1c (%), mean ± SD | | 8.67 ± 1.46 | 8.91 ± 1.46 |  | 7.92 ± 1.23 | 7.63 ± 1.47 |  | 8.86 ± 1.65 | 8.83 ± 1.56 |
| Baseline HbA1c (mmol/mol), mean | | 71 | 74 |  | 63 | 60 |  | 73 | 73 |
| Baseline eGFR (ml/min/1.73 m^2^), mean ± SD | | 86.78 ± 25.17 | 85.13 ± 27.46 |  | 79.22 ± 23.66 | 77.45 ± 24.21 |  | 97.00 ± 37.00 | 92.07 ± 39.83 |
|  | eGFR>90, n (%) | 2,228 (41.64) | 1,118 (39.65) |  | 281 (29.77) | 91 (27.25) |  | 174 (52.73) | 48 (45.28) |
|  | 60<eGFR≤90, n (%) | 2,375 (44.38) | 1,185 (42.02) |  | 3,071 (46.15) | 1,156 (42.10) |  | 99 (30.00) | 32 (30.19) |
|  | 45<eGFR≤60, n (%) | 604 (11.29) | 381 (13.51) |  | 984 (14.79) | 333 (12.13) |  | 44 (13.33) | 18 (16.98) |
|  | eGFR≤45, n (%) | 144 (2.69) | 138 (4.82) |  | 296 (4.45) | 119 (4.33) |  | 13 (3.94) | 8 (7.55) |
| eGFR change in year before index date, mean ± SD (ml/min/1.73 m^2^) | | -1.79 ± 13.44 | -1.57 ± 13.42 |  | -0.67 ± 12.06 | -0.54 ± 11.21 |  | -0.70 ± 12.33 | -0.97 ± 13.54 |
| Number of pre-index-date eGFR measurements, mean ± SD | | 6.39 ± 3.92 | 6.78 ± 4.34 |  | 6.67 ± 5.76 | 6.94 ±4.97 |  | 6.17 ± 3.85 | 6.66 ±5.61 |
| History of microvascular disease, n (%) | | 2,901 (54.21) | 1,570 (55.67) |  | 148 (15.68) | 62 (18.56) |  | 106 (32.12) | 38 (35.85) |
| History of cardiovascular disease, n (%) | |  |  |  |  |  |  |  |  |
|  | Ischemic heart disease | 1,602 (29.94) | 836 (29.65) |  | 424 (44.92) | 176 (52.69) |  | 79 (23.94) | 33 (31.13) |
|  | Heart failure | 547 (10.22) | 325 (11.52) |  | 60 (6.36) | 25 (7.49) |  | 15 (4.55) | 5 (4.72) |
|  | Atrial fibrillation | 196 (3.66) | 121 (4.429) |  | 56 (5.93) | 23 (6.89) |  | 2 (0.61) | 1 (0.94) |
|  | Stroke | 780 (14.58) | 432 (145.32) |  | 80 (8.47) | 37 (11.08) |  | 23 (6.97) | 9 (8.49) |
|  | Peripheral artery disease | 226 (4.22) | 161 (5.71) |  | 71 (7.52) | 32 (9.58) |  | 0 (0.00) | 0 (0.00) |
|  | Transient ischemic attack | 191 (3.57) | 103 (3.65) |  | 11 (1.17) | 6 (1.80) |  | 0 (0.00) | 0 (0.00) |
| Chronic kidney disease, n (%) | | 323 (6.04) | 243 (8.62) |  | 69 (7.31) | 29 (8.68) |  | 183 (55.45) | 62 (58.49) |
| Hypertension, n (%) | | 4,067 (76.00) | 2,151 (76.28) |  | 648 (68.64) | 228 (68.26) |  | 230 (69.70) | 75 (70.75) |
| Baseline status of frailty, n (%) | | 659 (12.32) | 381 (13.51) |  | 122 (12.92) | 43 (12.87) |  | 48 (14.55) | 19 (17.92) |
| Medication history in year prior to index date  Glucose-lowering agent, n (%) | |  |  |  |  |  |  |  |  |
|  | Metformin | NA^*^ | NA^*^ |  | NA^*^ | NA^*^ |  | NA^*^ | NA^*^ |
|  | SU | 2,622 (49.00) | 936 (33.19) |  | 573 (60.70) | 161 (48.20) |  | 163 (49.39) | 47 (44.34) |
|  | Meglitinide | 123 (2.30) | 57 (2.02) |  | 31 (3.28) | 7 (2.10) |  | 28 (8.48) | 13 (12.26) |
|  | DPP-4 inhibitor | 4,182 (78.15) | 2,126 (75.39) |  | 635 (67.27) | 199 (59.58) |  | 163 (49.39) | 55 (51.89) |
|  | Thiazolidinedione | 1,584 (29.60) | 641 (22.73) |  | 216 (22.88) | 56 (16.77) |  | 53 (16.06) | 18 (16.98) |
|  | GLP-1 receptor agonist | 152 (2.84) | 68 (2.41) |  | 16 (1.69) | 4 (1.20) |  | 13 (3.94) | 6 (5.66) |
|  | Acarbose | 1,195 (22.33) | 689 (24.43) |  | 141 (14.94) | 39 (11.68) |  | 46 (13.94) | 18 (16.98) |
|  | Insulin | 1,127 (21.06) | 717 (25.43) |  | 178 (18.86) | 64 (19.16) |  | 147 (44.55) | 50 (47.17) |
| Other medications, n (%) | |  |  |  |  |  |  |  |  |
|  | Loop diuretic | 337 (6.30) | 237 (8.40) |  | 95 (10.06) | 42 (12.57) |  | 28 (8.48) | 13 (12.26) |
|  | Thiazide diuretic | 113 (2.11) | 54 (1.91) |  | 88 (9.32) | 42 (12.57) |  | 15 (4.55) | 6 (5.66) |
|  | RASis | 3,510 (65.60) | 1,813 (64.29) |  | 648 (68.64) | 232 (69.46) |  | 172 (52.12) | 49 (46.23) |
|  | CCB | 1,050 (19.62) | 578 (20.50) |  | 442 (46.82) | 172 (51.50) |  | 84 (25.45) | 30 (28.30) |
|  | β-blocker | 1,891 (35.34) | 1,002 (35.53) |  | 452 (47.88) | 177 (52.99) |  | 91 (27.58) | 33 (31.13) |
|  | Aldosterone antagonists | 169 (3.16) | 102 (3.62) |  | 78 (8.26) | 36 (10.78) |  | 26 (7.88) | 12 (11.32) |
|  | Statins | 3,278 (61.26) | 1,655 (58.69) |  | 376 (39.83) | 131 (39.22) |  | 178 (53.94) | 54 (50.94) |
|  | Aspirin | 1,682 (31.43) | 865 (30.67) |  | 390 (41.31) | 143 (42.81) |  | 76 (23.03) | 27 (25.47) |
|  | Antiplatelet agent | 417 (7.79) | 243 (8.62) |  | 156 (16.53) | 77 (23.05) |  | 6 (1.82) | 1 (0.94) |
|  | Anticoagulant | 146 (2.73) | 81 (2.87) |  | 41 (4.34) | 22 (6.59) |  | 2 (0.61) | 1 (0.94) |
| SGLT2i category, n (%) | |  |  |  |  |  |  |  |  |
|  | Dapagliflozin | 2,271 (42.44) | 1,240 (43.97) |  | 587 (62.18) | 227 (67.96) |  | 106 (32.12) | 30 (28.30) |
|  | Empagliflozin | 3,080 (57.56) | 1,580 (56.03) |  | 357 (37.82) | 107 (32.04) |  | 224 (67.88) | 76 (71.70) |
| Quarter of SGLT2i initiation, n (%) | |  |  |  |  |  |  |  |  |
|  | 2^nd^ quarter, 2016 | 600 (11.21) | 335 (11.88) |  | 0 (0.00) | 0 (0.00) |  | 0 (0.00) | 0 (0.00) |
|  | 3^rd^ quarter, 2016 | 1,565 (29.25) | 794 (28.16) |  | 125 (13.24) | 44 (13.17) |  | 0 (0.00) | 0 (0.00) |
|  | 4^th^ quarter, 2016 | 969 (18.11) | 490 (17.38) |  | 284 (30.08) | 93 (27.84) |  | 85 (25.76) | 31 (29.25) |
|  | 1^st^ quarter, 2017 | 792 (14.80) | 429 (15.21) |  | 203 (21.50) | 75 (22.46) |  | 80 (24.24) | 23 (21.70) |
|  | 2^rd^ quarter, 2017 | 690 (12.89) | 395 (14.01) |  | 127 (13.45) | 44 (13.17) |  | 74 (22.42) | 24 (22.64) |
|  | 3^rd^ quarter, 2017 | 566 (10.58) | 273 (9.68) |  | 116 (12.29) | 41 (12.28) |  | 46 (13.94) | 15 (14.15) |
|  | 4^th^ quarter, 2017 | 169 (3.16) | 104 (3.69) |  | 89 (9.43) | 37 (11.08) |  | 45 (13.64) | 13 (12.26) |

Abbreviations: CCB, calcium channel blocker; CGMH, Chang Gung Memorial Hospital; DPP-4 inhibitor, dipeptidyl peptidase 4 inhibitor; eGFR, estimated glomerular filtration rate; GLP-1 receptor agonist, glucagon-like peptide 1 receptor agonist; HbA1c, hemoglobin A1c; NA, not applicable; NCKUH, National Cheng Kung University Hospital; NTUH, National Taiwan University Hospital; RASis, renin-angiotensin system inhibitors; SD, standard deviation; SGLT2is, sodium glucose cotransporter-2 inhibitors; SU, sulfonylurea; w, with; w/o, without.

^*^ This variable was not measured in the cohort and was not included in the estimation of propensity score and matching.

Note: All variables were comparable between patients with SGLT2i and metformin and those receiving SGLT2is alone after propensity score matching in each study institute, as supported by the standard mean difference less than 0.1.

**Supplementary Table 2.** Baseline characteristics of study cohort after propensity score matching in each institution, grouped by use of RASis with SGLT2is

| Characteristics | | CGMH | |  | NTUH | |  | NCKUH | |
| --- | --- | --- | --- | --- | --- | --- | --- | --- | --- |
|  |  | SGLT2i users w RASis | SGLT2i users  w/o RASis |  | SGLT2i users w RASis | SGLT2i users  w/o RASis |  | SGLT2i users w RASis | SGLT2i users  w/o RASis |
|  |  | n=5,190 | n=2,064 |  | n=1,098 | n=444 |  | n=368 | n=238 |
| Age (years), mean ± SD | | 61.03 ± 10.98 | 60.59 ± 11.54 |  | 63.51 ± 11.35 | 63.52 ± 11.62 |  | 60.14 ± 11.07 | 59.41 ± 11.69 |
| Male | | 3,072 (59.21) | 1,252 (60.66) |  | 648 (59.02) | 275 (61.94) |  | 206 (55.98) | 136 (57.14) |
| Baseline HbA1c (%), mean ± SD | | 8.72 ± 1.48 | 8.85 ± 1.47 |  | 8.01 ± 1.40 | 7.95 ± 1.40 |  | 8.81 ± 1.58 | 8.83 ± 1.53 |
| Baseline HbA1c (mmol/mol), mean | | 72 | 73 |  | 64 | 63 |  | 73 | 73 |
| Baseline eGFR (ml/min/1.73 m^2^), mean ± SD | | 81.72 ± 24.58 | 85.87 ± 26.08 |  | 79.86 ± 23.38 | 82.15 ± 24.63 |  | 98.96 ± 33.89 | 100.30 ± 35.11 |
|  | eGFR>90, n (%) | 1,750 (33.73) | 855 (41.42) |  | 341 (31.06) | 142 (31.98) |  | 212 (57.61) | 141 (59.24) |
|  | 60<eGFR≤90, n (%) | 2,434 (46.92) | 864 (41.86) |  | 524 (47.72) | 225 (50.68) |  | 113 (30.71) | 67 (28.15) |
|  | 45<eGFR≤60, n (%) | 770 (14.84) | 252 (12.21) |  | 181 (16.48) | 58 (13.06) |  | 33 (8.97) | 23 (9.66) |
|  | eGFR≤45, n (%) | 234 (4.51) | 93 (4.51) |  | 52 (4.74) | 19 (4.28) |  | 10 (2.72) | 7 (2.94) |
| eGFR change in year before index date, mean ± SD (ml/min/1.73 m^2^) | | -1.94 ± 12.86 | -1.50 ± 14.04 |  | -0.35 ± 11.19 | -0.67 ± 12.86 |  | -1.50 ± 13.32 | -0.89 ± 11.40 |
| Number of pre-index-date eGFR measurements, mean ± SD | | 6.60 ± 4.23 | 7.04 ± 4.78 |  | 6.16 ± 3.98 | 6.54 ±4.39 |  | 5.77 ± 3.29 | 6.00 ±3.82 |
| History of microvascular disease, n (%) | | 2,920 (56.28) | 1,198 (58.04) |  | 182 (16.58) | 76 (17.12) |  | 124 (33.70) | 74 (31.09) |
| History of cardiovascular disease, n (%) | |  |  |  |  |  |  |  |  |
|  | Ischemic heart disease | 1,755 (33.83) | 691 (33.48) |  | 481 (43.81) | 201 (45.27) |  | 107 (29.08) | 67 (28.15) |
|  | Heart failure | 654 (12.61) | 265 (12.84) |  | 75 (6.83) | 27 (6.08) |  | 26 (7.07) | 13 (5.46) |
|  | Atrial fibrillation | 230 (4.43) | 106 (5.14) |  | 66 (6.01) | 29 (6.53) |  | 14 (3.80) | 7 (2.94) |
|  | Stroke | 881 (16.98) | 342 (16.57) |  | 105 (9.56) | 32 (7.21) |  | 42 (11.41) | 23 (9.66) |
|  | Peripheral artery disease | 284 (5.47) | 123 (5.96) |  | 79 (7.19) | 31 (6.98) |  | 7 (1.90) | 5 (2.10) |
|  | Transient ischemic attack | 216 (4.16) | 89 (4.31) |  | 14 (1.28) | 5 (1.13) |  | 0 (0.00) | 0 (0.00) |
| Chronic kidney disease, n (%) | | 449 (8.65) | 166 (8.04) |  | 72 (6.56) | 25 (5.63) |  | 198 (53.80) | 122 (51.26) |
| Hypertension, n (%) | | 4,860 (93.68) | 1,735 (84.06) |  | 885 (80.60) | 290 (65.32) |  | 283 (76.90) | 155 (65.13) |
| Baseline status of frailty, n (%) | | 715 (13.78) | 316 (15.31) |  | 133 (12.11) | 52 (11.71) |  | 42 (11.41) | 27 (11.34) |
| Medication history in year prior to index date  Glucose-lowering agent, n (%) | |  |  |  |  |  |  |  |  |
|  | Metformin | 4,024 (77.56) | 1,555 (75.34) |  | 919 (83.70) | 375 (84.46) |  | 341 (92.66) | 220 (92.44) |
|  | SU | 2,208 (42.56) | 892 (43.22) |  | 683 (62.20) | 284 (63.96) |  | 251 (68.21) | 157 (65.97) |
|  | Meglitinide | 112 (2.16) | 48 (2.33) |  | 54 (4.92) | 18 (4.05) |  | 24 (6.52) | 14 (5.88) |
|  | DPP-4 inhibitor | 4,014 (77.37) | 1,575 (76.31) |  | 757 (68.94) | 294 (66.22) |  | 171 (46.47) | 114 (47.90) |
|  | Thiazolidinedione | 1,383 (26.66) | 572 (27.71) |  | 254 (23.13) | 112 (25.23) |  | 52 (14.13) | 30 (12.61) |
|  | GLP-1 receptor agonist | 143 (2.76) | 44 (2.13) |  | 19 (1.73) | 11 (2.48) |  | 23 (6.25) | 14 (5.88) |
|  | Acarbose | 1,284 (24.75) | 492 (23.84) |  | 175 (15.94) | 78 (17.57) |  | 65 (17.66) | 45 (18.91) |
|  | Insulin | 1,155 (22.26) | 507 (24.56) |  | 199 (18.12) | 77 (17.34) |  | 118 (32.07) | 76 (31.93) |
| Other medications, n (%) | |  |  |  |  |  |  |  |  |
|  | Loop diuretic | 441 (8.50) | 175 (8.48) |  | 103 (9.38) | 44 (9.91) |  | 24 (6.52) | 16 (6.72) |
|  | Thiazide diuretic | 130 (2.51) | 31 (1.50) |  | 95 (8.65) | 26 (5.86) |  | 17 (4.62) | 9 (3.78) |
|  | RASis | NA^*^ | NA^*^ |  | NA^*^ | NA^*^ |  | NA^*^ | NA^*^ |
|  | CCB | 1,275 (24.58) | 428 (20.74) |  | 575 (52.37) | 179 (40.32) |  | 119 (32.34) | 72 (30.25) |
|  | β-blocker | 2,211 (42.62) | 710 (34.40) |  | 560 (51.00) | 209 (47.07) |  | 125 (33.97) | 82 (34.45) |
|  | Aldosterone antagonists | 195 (3.76) | 83 (4.02) |  | 73 (6.65) | 41 (9.23) |  | 24 (6.52) | 17 (7.14) |
|  | Statins | 3,157 (60.85) | 1,239 (60.03) |  | 443 (40.35) | 187 (42.12) |  | 251 (68.21) | 162 (68.07) |
|  | Aspirin | 1,895 (36.53) | 642 (31.10) |  | 484 (44.08) | 179 (40.32) |  | 118 (32.07) | 68 (28.57) |
|  | Antiplatelet agent | 502 (9.68) | 186 (9.01) |  | 175 (15.94) | 71 (15.99) |  | 4 (1.09) | 2 (0.84) |
|  | Anticoagulant | 142 (2.74) | 78 (3.78) |  | 47 (4.28) | 18 (4.05) |  | 4 (1.09) | 2 (0.84) |
| SGLT2i category, n (%) | |  |  |  |  |  |  |  |  |
|  | Dapagliflozin | 2,183 (42.08) | 921 (44.62) |  | 673 (61.29) | 276 (62.16) |  | 121 (32.88) | 82 (34.45) |
|  | Empagliflozin | 3,005 (57.92) | 1,143 (55.38) |  | 425 (38.71) | 168 (37.84) |  | 247 (67.12) | 156 (65.55) |
| Quarter of SGLT2i initiation, n (%) | |  |  |  |  |  |  |  |  |
|  | 2^nd^ quarter, 2016 | 612 (11.80) | 248 (12.02) |  | 0 (0.00) | 0 (0.00) |  | 0 (0.00) | 0 (0.00) |
|  | 3^rd^ quarter, 2016 | 1,482 (28.57) | 590 (28.59) |  | 145 (13.21) | 54 (12.16) |  | 0 (0.00) | 0 (0.00) |
|  | 4^th^ quarter, 2016 | 932 (17.96) | 340 (16.47) |  | 337 (30.69) | 128 (28.83) |  | 89 (24.18) | 52 (21.85) |
|  | 1^st^ quarter, 2017 | 763 (14.71) | 315 (15.26) |  | 242 (22.04) | 99 (22.30) |  | 82 (22.28) | 60 (25.21) |
|  | 2^rd^ quarter, 2017 | 697 (13.43) | 276 (13.37) |  | 147 (13.39) | 60 (13.51) |  | 77 (20.92) | 49 (20.59) |
|  | 3^rd^ quarter, 2017 | 536 (10.33) | 205 (9.93) |  | 131 (11.93) | 62 (13.96) |  | 63 (17.12) | 42 (17.65) |
|  | 4^th^ quarter, 2017 | 166 (3.20) | 90 (4.36) |  | 96 (8.74) | 41 (9.23) |  | 57 (15.49) | 35 (14.71) |

Abbreviations: CCB, calcium channel blocker; CGMH, Chang Gung Memorial Hospital; DPP-4 inhibitor, dipeptidyl peptidase 4 inhibitor; eGFR, estimated glomerular filtration rate; GLP-1 receptor agonist, glucagon-like peptide 1 receptor agonist; HbA1c, hemoglobin A1c; NA, not applicable; NCKUH, National Cheng Kung University Hospital; NTUH, National Taiwan University Hospital; RASis, renin-angiotensin system inhibitors; SD, standard deviation; SGLT2is, sodium glucose cotransporter-2 inhibitors; SU, sulfonylurea; w, with; w/o, without.

^*^ This variable was not measured in the cohort and was not included in the estimation of propensity score and matching.

Note: All variables were comparable between patients with SGLT2i and RASis and those receiving SGLT2is alone after propensity score matching in each study institute, as supported by the standard mean difference less than 0.1.

**Figure S1**. Forest plot for 30%, 40%, and 50% eGFR reductions in matched SGLT2i users with or without background medication of sulfonylurea (SU) (negative exposure control) in overall study cohort from three health care delivery systems (CGMH, NCKUH, and NTUH) (intention-to-treat analysis)

Abbreviations: CGMH, Chang Gung Memorial Hospital; eGFR, estimated glomerular filtration rate; NCKUH, National Cheng Kung University Hospital; NTUH, National Taiwan University Hospital; SGLT2is, sodium glucose cotransporter-2 inhibitors; w, with; w/o, without.

**Figure S2-1**. Forest plot for 30%, 40%, and 50% eGFR reductions in matched SGLT2i users with or without background medication of metformin in overall study cohort from three health care delivery systems (CGMH, NCKUH, and NTUH) (strict on-treatment analysis)

Abbreviations: CGMH, Chang Gung Memorial Hospital; eGFR, estimated glomerular filtration rate; NCKUH, National Cheng Kung University Hospital; NTUH, National Taiwan University Hospital; NA, not available; SGLT2is, sodium glucose cotransporter-2 inhibitors; w, with; w/o, without.

**Figure S2-2**. Forest plot for 30%, 40%, and 50% eGFR reductions in matched SGLT2i users with or without background medication of RASis in overall study cohort from three health care delivery systems (CGMH, NCKUH, and NTUH) (strict on-treatment analysis)

Abbreviations: CGMH, Chang Gung Memorial Hospital; eGFR, estimated glomerular filtration rate; NCKUH, National Cheng Kung University Hospital; NTUH, National Taiwan University Hospital; NA, not available; RASis, renin-angiotensin system inhibitors; SGLT2is, sodium glucose cotransporter-2 inhibitors; w, with; w/o, without.

**Figure S2-3**. Forest plot for 30%, 40%, and 50% eGFR reductions in matched SGLT2i users with or without background medication of sulfonylurea (SU) (negative exposure control) in overall study cohort from three health care delivery systems (CGMH, NCKUH, and NTUH) (strict on-treatment analysis)

Abbreviations: CGMH, Chang Gung Memorial Hospital; eGFR, estimated glomerular filtration rate; NCKUH, National Cheng Kung University Hospital; NTUH, National Taiwan University Hospital; NA, not available; SGLT2is, sodium glucose cotransporter-2 inhibitors; w, with; w/o, without.

**Figure S3-1**. Forest plot of sensitivity analysis (where outcome was re-defined using at least two eGFR measurements) for 30%, 40%, and 50% eGFR reductions in matched SGLT2i users with or without background medication of metformin in overall study cohort from three health care delivery systems (CGMH, NCKUH, and NTUH) (intention-to-treat analysis)

Abbreviations: CGMH, Chang Gung Memorial Hospital; eGFR, estimated glomerular filtration rate; NCKUH, National Cheng Kung University Hospital; NTUH, National Taiwan University Hospital; SGLT2is, sodium glucose cotransporter-2 inhibitors; w, with; w/o, without.

**Figure S3-2**. Forest plot of the sensitivity analysis (where the outcome was re-defined using at least two eGFR measurements) for 30%, 40%, and 50% eGFR reductions in matched SGLT2i users with or without background medication of metformin in overall study cohort from three health care delivery systems (CGMH, NCKUH, and NTUH) (strict on-treatment analysis)

Abbreviations: CGMH, Chang Gung Memorial Hospital; eGFR, estimated glomerular filtration rate; NCKUH, National Cheng Kung University Hospital; NTUH, National Taiwan University Hospital; NA, not available; SGLT2is, sodium glucose cotransporter-2 inhibitors; w, with; w/o, without.

**Figure S4-1**. Forest plot of sensitivity analysis (where outcome was re-defined using at least two eGFR measurements) for 30%, 40%, and 50% eGFR reductions in matched SGLT2i users with or without background medication of RASis in overall study cohort from three health care delivery systems (CGMH, NCKUH, and NTUH) (intention-to-treat analysis)

Abbreviations: CGMH, Chang Gung Memorial Hospital; eGFR, estimated glomerular filtration rate; NCKUH, National Cheng Kung University Hospital; NTUH, National Taiwan University Hospital; NA, not available; RASis, renin-angiotensin system inhibitors; SGLT2is, sodium glucose cotransporter-2 inhibitors; w, with; w/o, without.

**Figure S4-2**. Forest plot of sensitivity analysis (where outcome was re-defined using at least two eGFR measurements) for 30%, 40%, and 50% eGFR reductions in matched SGLT2i users with or without background medication of RASis in overall study cohort from three health care delivery systems (CGMH, NCKUH, and NTUH) (strict on-treatment analysis)

Abbreviations: CGMH, Chang Gung Memorial Hospital; eGFR, estimated glomerular filtration rate; NCKUH, National Cheng Kung University Hospital; NTUH, National Taiwan University Hospital; NA, not available; RASis, renin-angiotensin system inhibitors; SGLT2is, sodium glucose cotransporter-2 inhibitors; w, with; w/o, without.
